
JOURNAL INFORMATION
==============================
NLM Title Abbreviation: South Med Rec
NLM Journal ID: 0027317

The Southern Medical Record

Publisher: Biomedical Journal Digitization Project

ARTICLE INFORMATION
==============================
PMCID: PMC9089538
Article version: 1
Subjects: Editorial and Miscellaneous

Book Notices

Print publication date: 1879 Feb 20
Volume: 9
Issue: 2
First page: 80
Last page: 80
License: This work is free of known copyright restrictions. For more information, please see PMC Back Issue Digitization.
License URL: https://creativecommons.org/publicdomain/mark/1.0/

==============================

### *BOOK NOTICES.*

PHYSIOLOGY. *Preliminary Course of Lectures* by JAMES T. WHITAKER, M.A. M.D., Professor of Physiology and Clinical Medicine at the Good Samaritan Hospital, etc.—On the influence of physiology upon practice; on the conservation of force; on the origin of life and the evolutions of its forms; and on protoplasm, bone, muscle, nerve and blood; 288 pages, illustrated.—Cincinnati, CHANCY R. MURRAY, 103 W. Sixth street, 1879.

This is an admirable work in which the popular and learned Prof. Whitaker presents "the foundation facts and principles upon which the steady edifice of physiology is built." It should be read by both student and practitioner.

DIFFERENTIAL DIAGNOSIS, *A Manual of the Comparative Semliology of the more important Diseases*, by F. DEHAVILLAND HALL, M.D., Assistant Physician to the Westminster Hospital, London. American edition with extensive additions.—Philadelphia, D. G. BRINTON, 115 Second street, 1879.

A volume of 200 pages in which diagnostic points of diseases are given in a well-defined, comprehensive plan; presented in tabular method from which comparisons may be most easily drawn, and correct conclusions deduced. It is an eminently practical and useful work.

SCRIBNER'S MONTHLY MAGAZINE is among the best and most handsomely illustrated literary journals in the world. It is filled with solid matter on religious and other topics, and has a corps of the best story writers in the country. It is the very magazine for the lady of the doctor's house. Scribner & Co., New York.

ST. NICHOLAS is the Child's Magazine, and is unrivaled in its beauty and the finish of its illustrations and stories. The boys and girls prize it as the prettiest and nicest gift that can be tendered. Subscribe for it. Scribner & Co., New York.

---

RECEIPTED. 1878.—N. B. Richards; A. F. Pound; A. A. Lyon; W. T. Kendall; J. B. Payne; Geo. Redwood; J. B. Bailey; B. F. Phipps; C. H. Smith; J. E. Pope; R. D. Jackson; W. N. Ames; J. M. Pinkston; S. L. Dement.—1878 and 1879: R. H. Warren; W. K. Jones; J. W. Butts.—1879: E. A. Cox; L. K. Branch; W. S, Glass; J· A. McCallum; D. S. Williams; L. V. Weathers; J. N. Wadsworth, 6 months; E. W. Flemming; D. B. Hamilton; A. A. Hill; B. R. Doster; T. S. Parham; S. G. Hart; F. S. Wisdom; J. W. Hill; J. A. Williams, to June; J. M. Stansill; D. R. Fox; L. B. Bouchelle; E. H. Sherrell; H. T. Shiell, 6 months; T. B. Calling; Robert Kells; W. C. Jones; L. P. Coyner; Preston Pond; John Riches; John Gerdine; A. R. Wellborn, 6 months; J. A. Hayes; J. E. Martin; S. M. Hogan; B. F. Darnell; M. K. Harrison; W. J. Reddett; J. S. Sanders; A. A. Lyon; J. S. Bacon; L. Alison; J. R. Johnson; H. A. Hurst; C. F. Rodgers; T. D. Penn; A. G. Hobbs; T. L. Quillian; J. W. Thomas; J. C. Moody; Dr. McMikal; A. B. McWhorter; J. G. McCrary; W. B. King; J. T. Stephens; J. A. Holloway. J. F. Pou, '78; J. M. Boring; J. T. Stephens.

---

### SPECIAL NOTICES.

☞The solubility of COATED PILLS is a matter of very great importance to the physician who may desire to prescribe those of Quinine and other medicines offensive to the taste and smell. Various experiments from different sources have demonstrated the fact that the Sugar-Coated pills prepared by WM. R. WARNER & Co. are the most soluble and reliable in this respect. Those containing Quinine are made of good material and full strength, as demonstrated by chemical analyses. These facts were established by LEROY M. YALE, M.D., of New York, and A. B. LYONS, Analytical Chemist, Detroit, and others. And the well-known reputation of the House is a sufficient guarantee.

☞CASCARA SAGRADO is the Spanish name of a tree indigenous to northern California and Oregon, known botanically as Rhamnus Purshiana. The bark of this tree has long been used on the Pacific coast among the Indians and old Spanish residents, as a remedy for habitual constipation and other disorders of the alimentary canal. Its reputation among the inhabitants of this seclion and the reports of physicians who have used it, has induced hundreds of physicians throughout the United States and Canada to test the new remedy in their practice, the need of a reliable corrective in the most intractable of all complaints, habitual constipation, being universally felt. The testimony of these physicians has been so pronounced in favor of the new remedy, and the resulting demand for it has been so great, that we have found it very difficult to obtain sufficient of the drug to supply our many orders. Many physicians write to us that they must have the Cascara at any price, as it has proved invalvable in their practice. PARKE, DAVIS & Co., Manufacturing Chemists, Cincinnati, Ohio.
